# Primary proprioceptive neurons from human induced pluripotent stem cells: a cell model for afferent ataxias

**DOI:** 10.1038/s41598-020-64831-6

**Published:** 2020-05-08

**Authors:** Chiara Dionisi, Myriam Rai, Marine Chazalon, Serge N. Schiffmann, Massimo Pandolfo

**Affiliations:** 1 Laboratory of Experimental Neurology, Université Libre de Bruxelles (ULB), 1070 Brussels, Belgium; 2 Laboratory of Neurophysiology, Université Libre de Bruxelles (ULB), 1070 Brussels, Belgium

**Keywords:** Induced pluripotent stem cells, Spinocerebellar ataxia

## Abstract

Human induced pluripotent stem cells (iPSCs) are used to generate models of human diseases that recapitulate the pathogenic process as it occurs in affected cells. Many differentiated cell types can currently be obtained from iPSCs, but no validated protocol is yet available to specifically generate primary proprioceptive neurons. Proprioceptors are affected in a number of genetic and acquired diseases, including Friedreich ataxia (FRDA). To develop a cell model that can be applied to conditions primarily affecting proprioceptors, we set up a protocol to differentiate iPSCs into primary proprioceptive neurons. We modified the dual-SMAD inhibition/WNT activation protocol, previously used to generate nociceptor-enriched cultures of primary sensory neurons from iPSCs, to favor instead the generation of proprioceptors. We succeeded in substantially enriching iPSC-derived primary sensory neuron cultures for proprioceptors, up to 50% of finally differentiated neurons, largely exceeding the proportion of 7.5% normally represented by these cells in dorsal root ganglia. We also showed that almost pure populations of proprioceptors can be purified from these cultures by fluorescence-activated cell sorting. Finally, we demonstrated that the protocol can be used to generate proprioceptors from iPSCs from FRDA patients, providing a cell model for this genetic sensory neuronopathy.

## Introduction

Human induced pluripotent stem cells (iPSCs) have been used for a decade to generate models of human diseases that recapitulate the pathogenic process as it occurs in affected cell types. Though the context of a cell culture dish is obviously different from the living animal or human being, many cell-autonomous processes can be reconstructed and analyzed in hiPSC-derived cultures, as well as interactions among different cell types that can be co-differentiated or co-cultured, even in 3D^1^.

A variety of neurological diseases are characterized by irregular, jerky movement or posture due to loss of proprioceptive sensory feedback, a disturbance called afferent ataxia^2^. The affected neurons are primary sensory neurons in the dorsal root ganglia (DRGs) relaying body position and movement (proprioception) to the central nervous system. Primary proprioceptive neurons may be the target of hereditary, developmental, degenerative, toxic, inflammatory and autoimmune pathology^2^. They are the neurons in DRGs with the largest cell bodies^3^, and express the neurotrophin receptor TrkC and the Ca^2+^ binding protein Parvalbumin (PV)^4^ plus additional markers, some of which, such as CDH13 and CRTAC1^5^, define specific subpopulations. Their axons form large myelinated fibers in peripheral nerves and in the dorsal columns of the spinal cord, where they ascend to the gracile and cuneate nuclei in the medulla. The pathway then continues to the thalamus and somatosensory cortex to provide the conscious perception of proprioception. Axonal branches from neurons innervating muscle spindles directly synapse on motor neurons providing the basis for the monosynaptic stretch reflex. Other branches go to Clarke’s column, where the spinocerebellar tracts originate. Accordingly, typical clinical consequences of pathology affecting proprioceptive neurons, in addition to afferent ataxia, include loss of deep tendon (stretch) reflexes and of conscious perception of position and movement of body parts, often associated with loss of perception of vibration^2^.

Proprioceptive neurons only represent about 7.5% of neurons in DRGs, the vast majority of DRG neurons being small pain-(nociceptors) and temperature-sensitive neurons and medium- and large-size mechanoreceptors. Nociceptors express the neurotrophin receptor TrkA, mechanoreceptors express the neurotrophin receptor TrkB, some also TrkC. All primary sensory neurons derive from a population of neural crest cells, induced by BMP, TGF-β and WNT signaling^6^, that is specified at the border between neural and non-neural ectoderm in the dorsal neural tube during gastrulation, then delaminates from the neural tube and migrates along a ventral pathway^7^. Coupled to migration, sensory neurogenesis occurs in two successive waves. The first wave is guided by the transcription factor Neurogenin 2^8^ and preferentially generates TrkB^+^ and/or TrkC^+^ mechanoreceptors and proprioceptors. The second wave is guided by Neurogenin 1^8^ and gives rise to small TrkA^+^ nociceptive neurons, but also to large TrkB^+^/TrkC^+^ neurons, as corroborated by the finding that in Neurogenin 2-null mice Neurogenin 1 is sufficient for these neurons to develop normally. Neurogenins initiate pan-neuronal programs but do not specify neuronal subtypes in the sensory lineage. This step requires the action of the Trk receptors and their cognate neurotrophins, which control cell survival, axonal growth, target innervation and the establishment of functional contacts between cells, and also regulate peptide and ion channel expression^9^. The RUNX family of transcription factors are key intrinsic mediators of these processes. RUNX1 and RUNX3, are expressed in DRGs after the neurogenins and act non-redundantly to promote sensory subtype identity. RUNX1 is expressed in TrkA^+^ nociceptive neurons, supporting their differentiation. RUNX3 promotes the segregation of a transient population co-expressing TrkB and TrkC into the TrkC^+^ proprioceptive and TrkB^+^ mechanoreceptive populations by repressing TrkB, both directly and indirectly, while maintaining TrkC expression. Cells that maintain high levels of expression of RUNX3 keep TrkC and become proprioceptors, whereas cells that reduce or lose RUNX3 expression become TrkB^+^/TrkC^+^ or TrkB^+^ mechanoreceptors. Interestingly, RUNX3^−/−^ mice have loss of proprioceptive neurons and severe limb ataxia.

So far, efforts to differentiate human iPSCs into primary sensory neurons have succeed in either generating an unselected mixed neuronal population with only a small minority of proprioceptors or have focused on generating nociceptors to study pain disorders^10^. Here we report the use of a combination of small molecules and trophic factors to modulate the pathways involved in the differentiation of DRGs to obtain primary sensory neuronal cultures from human iPSCs that are highly enriched for proprioceptors, which can be further purified from these cultures by fluorescence-activated cell sorting (FACS).

We also demonstrate that this protocol is appropriate for disease modeling, as it can be successfully applied to iPSCs from patients with Friedreich ataxia (FRDA), an autosomal recessive multisystem disorder with prominent neurological manifestations characterized by early and severe loss of large, mostly proprioceptive sensory neurons in DRGs^11^, causing afferent ataxia, loss of deep tendon reflexes and loss of conscious proprioception. FRDA is also associated with cerebellar and pyramidal degeneration, mostly responsible for neurological disease progression^12^, as well as hypertrophic cardiomyopathy, skeletal abnormalities and carbohydrate intolerance. In almost all cases, the underlying genetic mutation is the hyperexpansion of a GAA repeat in the first intron of the *FXN* gene, encoding frataxin (FXN). The expanded GAA repeats promote chromatin condensation and disrupt *FXN* mRNA transcription^13^, leading to markedly reduced FXN levels in affected individuals. Reduced FXN levels impair the mitochondrial biogenesis of iron-sulfur (Fe/S) clusters^14^, leading to loss of activity of multiple Fe/S enzymes, mitochondrial dysfunction, and altered iron metabolism. Interestingly, at least in the mouse, proprioceptors express high levels of FXN, accounting for most FXN found in DRGs^15^, a possible clue to their specific vulnerability in FRDA. We^16–18^ and others^19–21^ have characterized the genetic, epigenetic and biochemical phenotype of iPSC-derived “default” neurons, close to immature cortical neurons, showing that they recapitulate essential aspects of FRDA pathogenesis that are at least partially corrected by restoring FXN levels. Although in some studies on FRDA iPSC neural differentiation has been cued toward a neural crest-primary sensory neuron fate^22^, or even to primary sensory neurons^19^, the lack of a specific protocol to obtain primary proprioceptive neurons has so far prevented deeper investigations on the pathological process affecting these cells in FRDA and on their specific vulnerability.

## Materials and Methods

### Induced pluripotent stem cell culture

iPSCs were obtained by reprogramming from human fibroblasts from two healthy controls (HEL46.11, HEL24.3) and three FRDA patients (HEL135.2, ULBi004FA4, ULBi005FA1). Participating individuals provided written informed consent and underwent a skin biopsy according to a protocol approved by the institutional Ethics Committee of Erasme Hospital (P2008.313) entitled “Generation of Cellular Models of Neurological Diseases”.

iPSCs were cultured under feeder-free conditions, in Essential 8 Medium (E8, Thermo Fisher Scientific, Cat. No.: A1517001) on Matrigel-coated tissue culture plates (Corning, Cat. No. 356231; 0.05 mg/ml Matrigel solution in DMEM/F12 medium). Cells were fed daily and passaged every 3 days using 0.5 mM EDTA.

### Differentiation of iPSCs into proprioceptor-enriched primary sensory neuron cultures

We modified the dual-SMAD inhibition/WNT activation protocol^10^ to differentiate primary sensory neurons from human iPSCs to favor differentiation into TrkC^+^ proprioceptors rather than TrkA^+^ nociceptors as in the original report.

iPSCs were plated as single cells on Matrigel treated dishes (0.5 mg/ml Matrigel solution in DMEM/F12 medium) at a density of around 20.000 cells/cm^2^ in E8 medium supplemented with 10 µM ROCK Inhibitor (Y-27632 dihydrochloride, Sigma, Cat. Y0503). The day after, the spent medium was replaced with fresh E8 without Y-27632, and cells were allowed to proliferate until 60–80% confluency (usually reached in 48 hours after seeding). To initiate sensory neuron differentiation, medium was replaced with Essential 6 Medium (E6 Medium, Thermo, Cat. A1516401) supplemented with 100 nM LDN193189 and 10 μM SB431542 (Day 1). E6 medium is equal to E8 minus bFGF and TGFβ proteins, which inhibit differentiation. LDN193189 and SB431542 were added until Day 5 of differentiation. Starting on Day 2, other three small molecules were added: 3 μM CHIR99021 was added from Day 2 to Day 7, while 10 μM DAPT and 9 μM SU5402 where added until Day 8. All inhibitor factors were purchased from STEMCELL Technologies. Cells were fed daily and medium was gradually switched from E6 medium to N2-A medium, starting on Day 4, according to the following schedule: Day 4–5 (75% E6, 25% N2-A), Day 6 (50% E6, 50% N2-A), Day 7–8 (25% E6, 75% N2-A). N2-A medium consists of Neurobasal-A medium (Thermo, Cat. 10888022), supplemented with 1% N2 (100×, Thermo, Cat. 17502001) and 1% GlutaMAX (100×, Thermo, Cat. 35050061). On Day 9, cells were passaged on new Matrigel coated plates to promote maturation and enhance neural survival. At this stage, cells used to be weakly attached to the substrate, so it was possible to gently dissociate them mechanically, without the need of Accutase or other enzymatic solutions, with minimum cellular stress and high survival rate after passaging. The spent medium was removed, cells were rapidly washed once in PBS and fresh medium was added directly to the plate. Cells were mechanically dissociated by gently dispensing the medium a few times against the culture surface and directly transferred into the new plate. It is important for cells to be re-plated at a high confluency to enhance long term survival. The splitting ratio should be adjusted for each line, according to the survival and proliferative rate observed in the first part of the protocol. However, if cells displayed a good confluency at this stage, it was possible to keep them in the original plate, with no mechanical stress or change in their original interactions and organization; this was observed to promote optimal cell survival and health.

**Figure 1:**
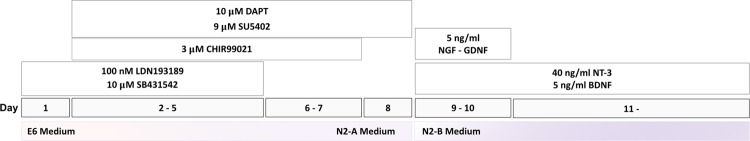
Scheme of the differentiation protocol.

Starting on Day 9, cells were fed in N2-B medium, consisting of Neurobasal-A medium supplemented with 1% N2, 1% B27 (Thermo, Cat. 17504001), 1% GlutaMAX, 1% MEM Non-Essential Amino Acids (Thermo, Cat. 11140050) and 0.1% β-mercaptoethanol. 40 ng/ml NT3 and 5 ng/ml BDNF were added from this point on, while 5 ng/ml Nerve Growth Factor (NGF) and Glial-Derived Neurotrophic Factor (GDNF) were added until Day 10. Neurotrophic factors were obtained from STEMCELL Technologies. Cells were fed by replacing 75% of medium containing neurotrophins every 2 days. Addition of neurotrophic factors stalls neural proliferation and stimulates a rapid maturation towards a pseudo-unipolar phenotype. The differentiation protocol is summarized in Fig. 1.

### Quantitative Real-Time PCR

Cells were harvested at day 2 (first days of differentiation), day 6 (tubular structures), day 8 (migrating cells), day 9 (passage into new plate), and after 3, 5, 7 and 12 days of treatment with neurotrophic factors and processed for total RNA extraction using the RNeasy Mini RNA Kit (Qiagen), following manufacturer’s instructions. Quality of RNA was examined on NanoDrop ND-1000 Spectrophotometer (Isogen) with a A_260/280_ ratio of around 2.0.

Up to 300 ng of total RNA was reverse transcribed using M-MLV Reverse Transcriptase (Thermo, Cat. 28025013) and random primers for cDNA synthesis, according to manufacturer’s protocol. Gene expression was quantified by quantitative reverse transcription PCR (RT-qPCR) on a 7500 Fast RT-PCR System (Applied Biosystem) using Power SYBR Green Master Mix (Thermo, Cat. 4367659). Relative RNA levels were analyzed by the threshold cycle (2^−ΔΔCT^) method normalized to GAPDH expression in iPSCs cells. Expression values are log_2_ of the fold change, with error bars representing standard deviation of mean (SD). Primer sequences are detailed in Supplemental Table 1.

Average log2 differences in PV, RUNX1, RUNX3 and FXN expression at day 0, 9 and 20 and average log2 relative changes from day 0 to day 20 were compared between control and FRDA lines by unpaired t-test with Welch’s correction using GraphPad Prism Software (GraphPad Software, San Diego, CA). Only results with P < 0.05 are reported.

### Immunofluorescence (IF)

Cells grown on Matrigel-coated glass coverslips were fixed in 3.6% paraformaldehyde (PFA, Sigma) for 10 minutes at room temperature and carefully washed twice in PBS after incubation. Membranes were permeabilized with ice cold 0.1% Triton X-100 in PBS for 10 minutes. After washing twice in PBS, non-specific binding sites were blocked with 10% Normal Donkey Serum (NDS, Abcam, Cat. ab7475) for 30 minutes at room temperature. Cells were then incubated with primary antibodies in 5% NDS overnight at 4 °C. Following three 5-minute washes in PBS, cells were incubated with secondary antibodies in 5% NDS for 1 hour at room temperature, protected from light. Nuclei were stained with Hoechst 33342 (1 μg/ml, Thermo, Cat. H3750) for 10 minutes at room temperature, protected from light. Samples were then mounted on microscope slides using FluorSave Reagent (Calbiochem, Cat. 345789). Images were acquired using ZEISS Axio Zoom.V16 Microscope or ZEISS AxioImager Z1 (Zeiss, Oberkochen, Germany), and processed using Zeiss ZEN 2.6 Blue Microscopy Software.

Primary antibodies used for immunofluorescence analysis included: SOX10, (1:50, R&D, Cat. MAB2864), Brn3a (1:500, Millipore, Cat. AB5945), Peripherin (1:500, Abcam, Cat. ab99942), Tubulin β-III (1:700, BioLegend, Cat. 801201), TrkA, (1:250, Abcam, Cat. ab76291), TrkB (1:250, Abcam, Cat. ab18987), TrkC (1:250, Abcam, Cat. ab43078 and 1:50, Santa Cruz, Cat. WW6), PV (1:250, Abcam Cat. ab11427). Secondary antibodies included: Donkey Anti-Mouse IgG Alexa Fluor 594 (1:1000, Abcam, Cat. ab150118) and Donkey Anti-Rabbit IgG Alexa Fluor 488 (1:1000, Abcam, Cat. ab150073).

The open source Cell Profiler software (v. 3.1.9) was used to obtain quantitative estimates of the proportion of specifically labeled cells in IF images. Total number of cells was estimated by Hoechst 33342 nuclear staining. Low magnification images (100x to 179x) were preferentially analyzed, as they provide an overall unbiased assessment of the proportion of positive cells in culture plates. Images for each investigated marker (TrkA, TrkB, TrkC, PV) were analyzed from multiple fields from at least two separate differentiations.

### Electrophysiology

For electrophysiological recordings, individual culture slides from control lines at different stages of maturation (day 7, 10, 12 of treatment with neurotrophic factors) were transferred to a thermoregulated (30–32 °C) chamber, maintained immersed and continuously superfused at a rate of 1,5–2 ml/min with oxygenated artificial cerebrospinal fluid (aCSF) containing (in mM): NaCl 127, KCl 2.5, NaH_2_PO_4_ 1.25, MgCl_2_ 1, NaHCO_3_ 26, D-glucose 10, CaCl_2_ 2, bubbled with 95% O_2_ and 5% CO_2_ at a pH of 7.3 (300–316 mOsm). Patch clamp experiments were performed in whole cell configuration on individual neurons, identified with a 63x water immersion objective from Zeiss Axioskop microscope (Axioskop 2FS Plus; 140 Zeiss, Oberkochen, Germany) with an infrared CCD camera (X-ST70CE, Hamamatsu Photonics KK, Hamamatsu, Japan). Cells were selected based on their size and morphology; as proprioceptive neurons are reported to be among the largest sensory neurons in DRGs, only large and pseudo-unipolar neurons were patched.

Borosilicate-glass patch electrodes [4–6 MΩ, (Hilgenberg GmbH, Malsfeld, Germany)] were filled with a solution containing biocytin 0.5% (Sigma-Aldrich, Cat. B4261) and (in mM): KMeSO_4_ 125, KCl 12, CaCl_2_ 0.022, MgCl_2_ 4, HEPES 10, EGTA 0.1, Na_2_-phosphocreatine 5, Mg_2_-ATP 4, Na_2_-GTP 0.5 (pH of 7.2, 292 mOsm). Currents were recorded using an EPC-10 patch clamp amplifier (HEKA, Lambrecht, Germany) and PatchMaster acquisition software (HEKA).

Cells were first recorded in voltage-clamp mode at holding potential of −60 mV with a gain of 2 mV/pA and low-pass filtered at 2.9 kHz. Signals were sampled at 20 kHz. Passive membrane properties (Capacitance (Cm, pF), Membrane resistance (Rm, MΩ), Membrane time constant (τ, ms)) and series resistances were extracted from current traces recorded in response to a hyperpolarizing voltage pulse (200 ms) of −10 mV from holding potential. Ten sweeps were averaged to remove noise.

Then, in current-clamp mode, cell excitability was investigated by setting the resting membrane potential at −60 mV and injecting 1 s depolarizing steps (from 0 to 100 pA in 10 pA increments). Signals were sampled at 10 kHz with a gain of 2 mV/pA. To measure the neuron resting membrane potential (RMP), the potential fluctuations over the duration of the step at 0 pA of injected current were averaged off-line. For cells recorded with spontaneous action potentials (APs), the RMP was measured manually by the value of neuron potential at 0.02 s before the first action potential of the step at 0 pA. Series resistance was not compensated during the recordings and membrane potential values were corrected off-line with a liquid junction potential of 6.6 mV. If access resistance exceeded 35 MΩ and changed more of 25% between the beginning and the end of the recording, the neuron was discarded. The series resistance averaged for all recordings is of 27,32 ± 1,331 MΩ.

Analysis of passive properties and excitability of patched neurons were performed with IgorPro 6.3 software (WaveMetrics, Portland, USA) using Patcher’sPower Tools, NeuroMatic plugins and Microsoft Excel software. Results are expressed as mean ± SEM.

When possible, confirmation of morphology and identity of recorded neurons was assessed with biocytin-TrkC double immunostaining (N = 7). For this purpose, neurons were filled with biocytin, directly included in the pipette internal solution at a concentration of 0.5%. In order to confirm their identity and stage of maturation, they were then double stained for biocytin and TrkC. Following patch clamp experiments, culture slides were fixed in 3.6% PFA, permeabilized with 0.1% Triton X-100 and blocked with 10% NDS, as previously described. They were incubated overnight at 4 °C with anti-TrkC primary antibody in 5% NDS. The following day cells were washed three times in PBS for 5 minutes, then incubated with anti-rabbit secondary antibody (1:1000) and streptavidin-NL557 (1:2000) in 5% NDS for 2 hours at room temperature, protected from light. Culture slides were washed three times in PBS and mounted with FluorSave Reagent for imaging. Z-stack sections were acquired using ZEISS Axio Zoom.V16 Microscope and processed with Zeiss ZEN 2.6 Blue Microscopy Software.

### Flow cytometry

Neurons were collected after one week of treatment with neurotrophins using StemPro Accutase and gently triturated with a 1000 μl pipette into a single cell suspension. Cell clumps were removed by filtration through a 70 μm strainer. Cells were counted and spun at 200 g for three minutes. The cell pellet was resuspended at a concentration of 10^7^ cells/ml in cold Flow Cytometry (FC) Staining Buffer 1 × (R&D, Cat. FC001) and transferred into a protein low-binding tube. A small aliquot of 2 × 10^5^ cells was transferred into another tube for use as negative control, without staining with any antibodies. The rest of cell suspension was split into three different tubes (around 10^6^ cells each), for staining of Trk receptors: the first sample was incubated with an anti-TrkA antibody conjugated with Alexa Fluor 405 (1 μg/10^6^ cells, R&D, Cat. FAB1751RV), the second with an anti-TrkB antibody conjugated with Alexa Fluor 488 (1 μg/10^6^ cells, R&D, Cat. FAB3971G) and the third with an anti-TrkC antibody conjugated with Phycoerythrin (10 μl/10^6^ cells, R&D, Cat. FAB373P) for 1 hour at +4 °C in constant low speed rotation, protected from light. At the end of incubation, cells were spun at 300 g for 3 minutes at 4 °C, washed with 500 μl of FC Staining Buffer and resuspended in 200 μl of buffer. TrkA^+^, TrkB^+^ and TrkC^+^ cells were quantified in a BD FACS Aria II System (BD Bioscience). The flow cytometer was calibrated using the negative control without antibody and parameters were adjusted to remove any contribution by unlabeled cells. DAPI filter sets were used for detection of Alexa Fluor 405 emission spectra, FITC filters for detection of Alexa Fluor 488 and the PE channel for detection of Phycoerythrin signals. Results were analyzed using FlowJo. TrkC^+^ cells were sorted and directly dissolved in RTL buffer for subsequent RNA extraction and RT-qPCR analysis: for each experimental sample (n = 4), around 10^5^ events were collected. Efficiency of sorting and nature of sorted cells were assessed by comparison of Trk receptors and proprioceptor marker expression between sorted cells and original cultures (differentiated neurons obtained from same plates used for FACS).

## Results

### iPSC differentiation into proprioceptor-enriched primary sensory neuron cultures

Figure 1 shows a summary of the steps of the differentiation protocol. The process of iPSC differentiation in culture recapitulated the *in vivo* DRG development. Initially, iPSCs proliferated in a uniform confluent layer, without any significant morphological changes. Between day 5 and 6, cells rapidly formed regular tubular aggregates where they were tightly connected. On day 7, cells with the morphology of neural precursors started migrating out of the tubular aggregates, which rapidly disappeared in the following two days, forming a uniform layer. At this stage (day 9), cells were passed into a new plate and allowed to mature in the presence of neurotrophic factors. In this second phase, cells showed rapid morphological changes, from bipolar, to bell-shaped to pseudo-unipolar neurons. Such phenotypical maturation was coupled with the organization of neurons in clusters and with the generation of a tight network of neurites.

**Figure 2:**
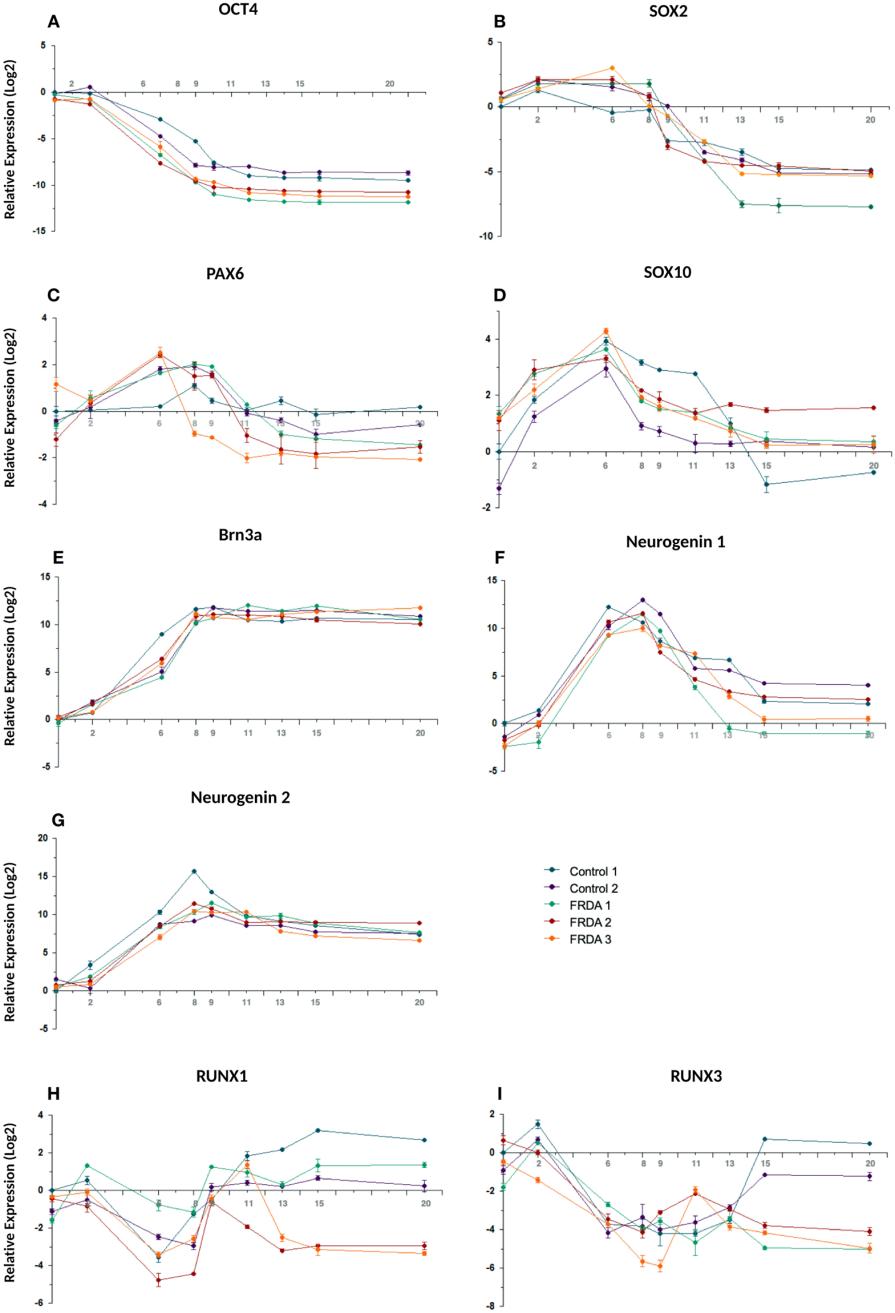
Time course of the expression (log2 scale) of nine transcription factors during iPSC differentiation to sensory neurons in two control and three FRDA lines. mRNA levels were determined by quantitative RT-PCR in duplicate experiments and normalized to levels in control iPSCs.

**Figure 3:**
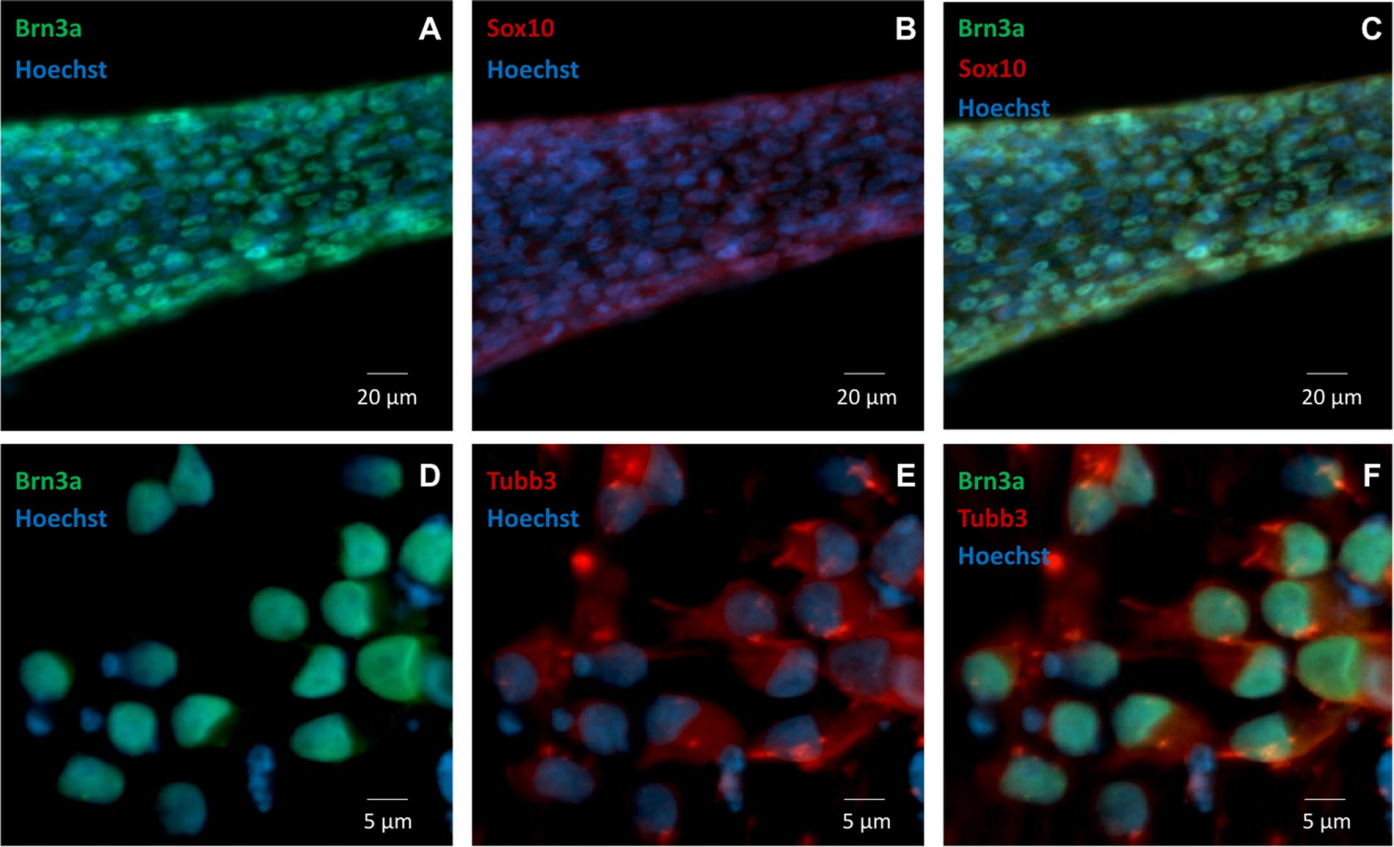
(**A–C**) SOX10 (green) and BRN3A (red) expression by IF in tubular aggregates of neural precursors at day 5. Nuclei are shown in blue (Hoechst). **(D–F)** BRN3A (green) TUBB3 (red) expression by IF in differentiated sensory neurons at day 15. Nuclei are shown in blue (Hoechst).

**Figure 4:**
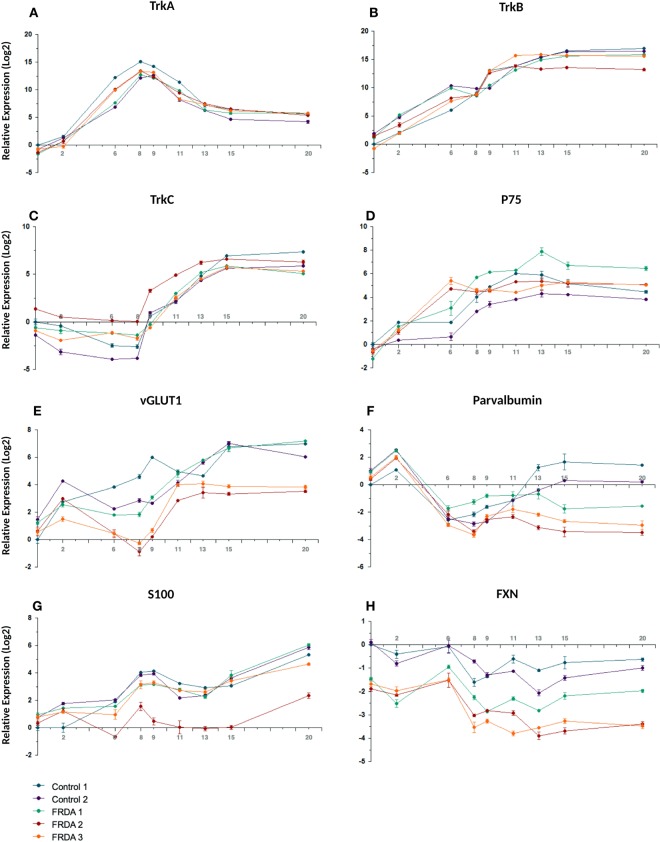
Time course of the expression (log2 scale) of Trk receptors, p75, vGLUT1, PV, S100 and FXN during iPSC differentiation to sensory neurons in two control and three FRDA lines. mRNA levels were determined by quantitative RT-PCR in duplicate experiments and normalized to levels in control iPSCs.

**Figure 5:**
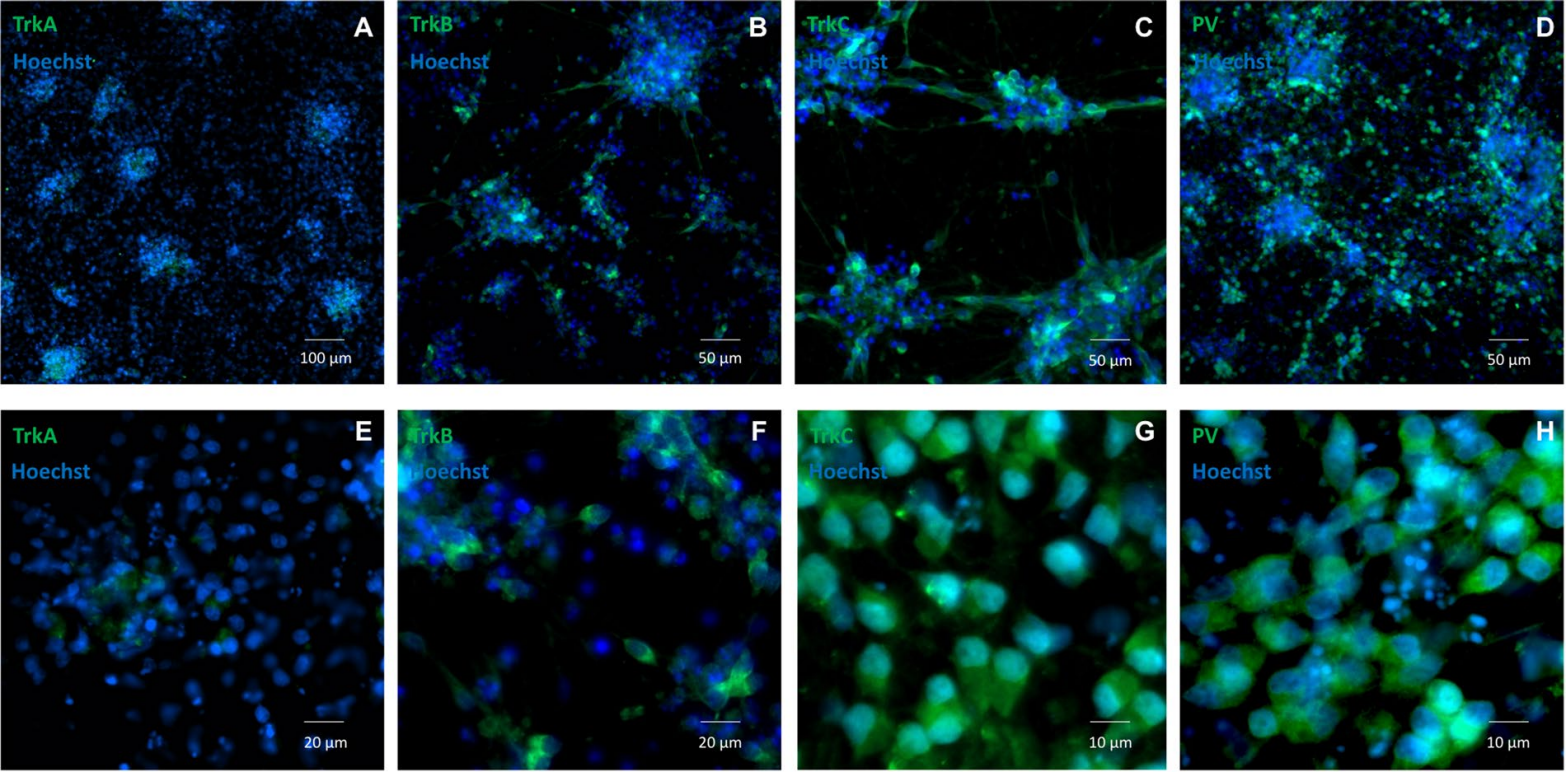
(**A,E**) TrkA; **(B,F)** TrkB; **(C,G)** TrkC; (**D,H**) PV expression by IF in differentiated sensory neurons after 7 days of exposure to neurotrophic factors (day 15 of differentiation). Staining for all antibodies is in green. Nuclei are shown in blue (Hoechst).

The timing and efficiency of the process were investigated by qRT-PCR and by IF analysis of the expression of transcription factors involved at different stages of sensory neurogenesis and of other markers of pluripotent cells, neural crest cells, developing and mature sensory neurons. At the beginning of the differentiation process cells rapidly lost the expression of the pluripotent marker OCT4, retaining the marker for multipotent neural stem cells SOX2 until day 9 (Fig. 2A,B). At the same time the human neuroectoderm marker PAX6 was only transiently and weakly induced, indicating that the treatment mostly generated cells of the sensory lineage (Fig. 2C). Expression of the neural crest marker SOX10 peaked at day 6 (16-fold compared to iPSC level) then rapidly declined, returning to iPSC level or lower at day 9, indicating a transient transition through a neural crest phenotype (Fig. 2D). BRN3A, marker of developing and mature sensory neurons, showed a strong induction (3–5 × 10^3^-fold), plateauing around day 8–9 when SOX10 was declining (Fig. 2E). Figure 3A–C shows co-expression of BRN3A and SOX10 in tubular structures at day 6, Fig. 3D–F shows persisting BRN3A expression in differentiated sensory neurons labeled with the specific neuronal marker TUBB3. Neurogenin 1 and Neurogenin 2 expression peaked between day 6 and 9, with a robust 3–5×10^3^-fold increase compared to iPSC levels, without clearly showing the two distinct waves occurring *in vivo* (Fig. 2F-G). Their expression declined when neurotrophic factors were added, leading to the final sensory neuron differentiation. Addition of neurotrophins and expression of Trk receptors (Figs. 4 and 5) marked the final stage of sensory neurogenesis. The brief (day 9–10) exposure to low levels (5 ng/ml) of NGF in the second part of the protocol transiently sustained the differentiation and survival of nociceptors, as also indicated by moderate induction of RUNX1 (Fig. 2H), which was not significantly different between FRDA and control lines. However, nociceptors constituted a minority of the differentiated sensory neurons and they were progressively lost after the removal of NGF from the culture medium, as indicated by a progressive reduction in the expression of TrkA (Figs. 4A, 5A,E), which was eventually 10^3^-fold lower in differentiated cultures than at the beginning of final sensory neuron differentiation (day 9). The expression of TrkB continued instead to increase until day 15, then remained stable despite the low concentration of BDNF in the culture (5 ng/ml) (Figs. 4B and 5B,F), probably due to its tight association with TrkC. Contrary to the other Trk receptors, TrkC was already robustly expressed in iPSCs, then showed a slight (2–8-fold) decrease in the first part of differentiation. This is consistent with the observation that TrkC is among the earliest markers of sensory neurogenesis *in vivo* and is already expressed by migrating neural crest and neural progenitor cells. TrkC expression increased again in the final differentiation stage, when a high concentration of NT-3 (40 ng/ml) was used, to up to 100-fold its iPSC level (Figs. 4C and 5C–G).

**Figure 6:**
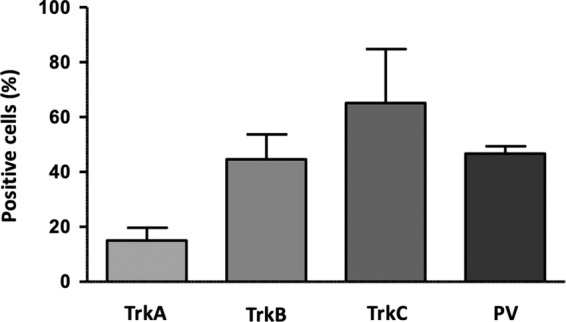
Bar graph showing the percentage of TrkA^+^, TrkB^+^, TrkC^+^ and PV^+^ cells by quantification of IF images of cultures after 7 days of exposure to neurotrophic factors (day 15 of differentiation).

Quantitative analysis of IF images at this stage showed that about 70% of cells expressed TrkC, about 50% expressed TrkB, and about 15% expressed TrkA (Fig. 6). The proportion of TrkA expressing cells is likely an overestimate, because it was obtained by analyzing rare fields containing positive cells, as evident in low magnification images (Fig. 5A). The fact that TrkB^+^ and TrkC^+^ cells add to >100% indicates the presence of double positives, which include some mechanoreceptors and some still not finally committed precursors of proprioceptors.

P75^NTR^, member of the tumor necrosis factor receptor family, is a low affinity receptor for all neurotrophins expressed on neural crest cells and co-expressed with Trk receptors in sensory neurons. It functionally collaborates with Trk receptors to enhance responses to preferred Trk ligands, to reduce responses to non-preferred ligands, and to facilitate apoptosis resulting from neurotrophin withdrawal. The expression of p75^NTR^ was progressively induced throughout all phases of the treatment and persisted in mature neurons (Fig. 4D). Specific markers of proprioceptive neurons that were induced in the final stage of differentiation included the transcription factor RUNX3 (Fig. 2I), vGLUT1 and PV (Figs. 4F and 5D,H). RUNX3 expression was relatively high in iPSCs, then it declined during neural crest specification and increased again in the second part of the protocol during the final specification and maturation of proprioceptive neurons. At day 20, the induction of RUNX3 compared to iPSC levels (day 0) was significantly lower in FRDA cells (P = 0.007; Fig. 2I). Similarly, PV expression was relatively high in iPSCs (day 0), declined during neural crest specification and increased again in the final differentiation stage. Though there was no significant difference in PV induction between control and FRDA cells, the average PV expression was lower in FRDA cells at day 20 (P = 0.0349; Fig. 4F). By quantitative assessment of IF images PV^+^ cells reached about 50% (Fig. 6), providing an estimate of the proportion of proprioceptors in our finally differentiated cultures, a 4–5-fold enrichment compared to the physiological composition of DRG neurons.

S100, a marker for glia and satellite cells in DRGs, showed progressive induction with a transient small dip at the beginning of the final sensory neuronal differentiation (Fig. 4G). FXN expression showed a similar mild decline in control as well as FRDA lines after neural crest induction around day 6 (Fig. 4H). However, throughout the differentiation process FRDA iPSCs expressed 3–4 fold lower levels of FXN mRNA than control lines (P = 0.002 at day 0; P = 0.006 at day 8; P = 0.037 at day 20), confirming the continued inhibition of *FXN* gene expression by the expanded GAA repeats.

Overall, we could successfully mimic the initial *in vivo* differentiation of primary sensory neurons, then strongly enrich for cells expressing proprioceptive neurons markers in the final phase of neuronal type determination.

### FACS purification

**Figure 7:**
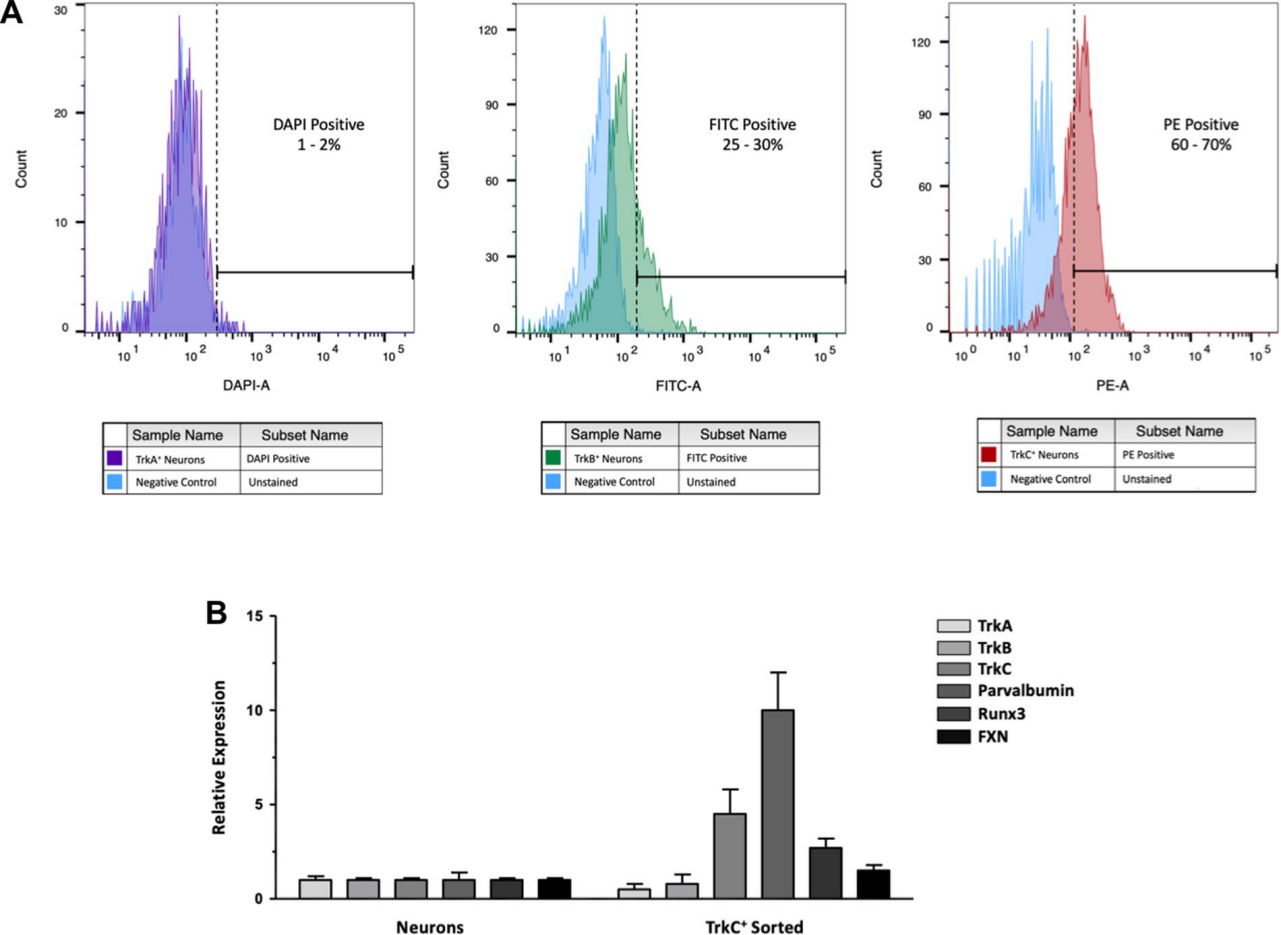
(**A**) Expression of Trk receptors assessed using flow cytometry on cultures after 7 days of exposure to neurotrophic factors (day 15 of differentiation). Unstained neurons were used as negative control (light blue histograms). In each panel, the average of positive cells observed for each marker in all trials performed (n = 4) is reported: TrkA^+^ neurons represented the 1–2% of differentiated cultures (violet histogram), TrkB^+^ the 25–20% (green histogram), while TrkC^+^ cells were the 60–70% (red histogram) in analyzed samples. Channels used for detection included: DAPI (Alexa Fluor 405), FITC (Alexa Fluor 488), PE (Phycoerythrin). (**B**) Fold-difference of Trk receptor and proprioceptor marker expression by quantitative RT-PCR analysis in sorted cells vs. starting sensory neurons cultures.

By FACS analysis, after one week of treatment with neurotrophic factors (day 15) TrkC^+^ cells represented 60–70% of differentiated cultures, TrkB^+^ cells 25–30%, while TrkA^+^ cells were only 1–2% (Fig. 7A). These findings are in line with the quantification of IF images of differentiated cultures, considering that the latter overestimated TrkA^+^ cells.

Quantitative RT-PCR analysis of FACS purified TrkC^+^ cells showed a marked enrichment for TrkC, PV and RUNX3, with a concomitant reduction in TrkB and TrkA (Fig. 7B), indicating that sorted cells were mostly single positive for TrkC, with a proprioceptive identity. Interestingly, purified TrkC^+^ cells also had a higher FXN expression (Fig. 7B), in line with the observation that this protein is enriched in proprioceptors.

### Functional characterization of differentiated neurons

**Figure 8:**
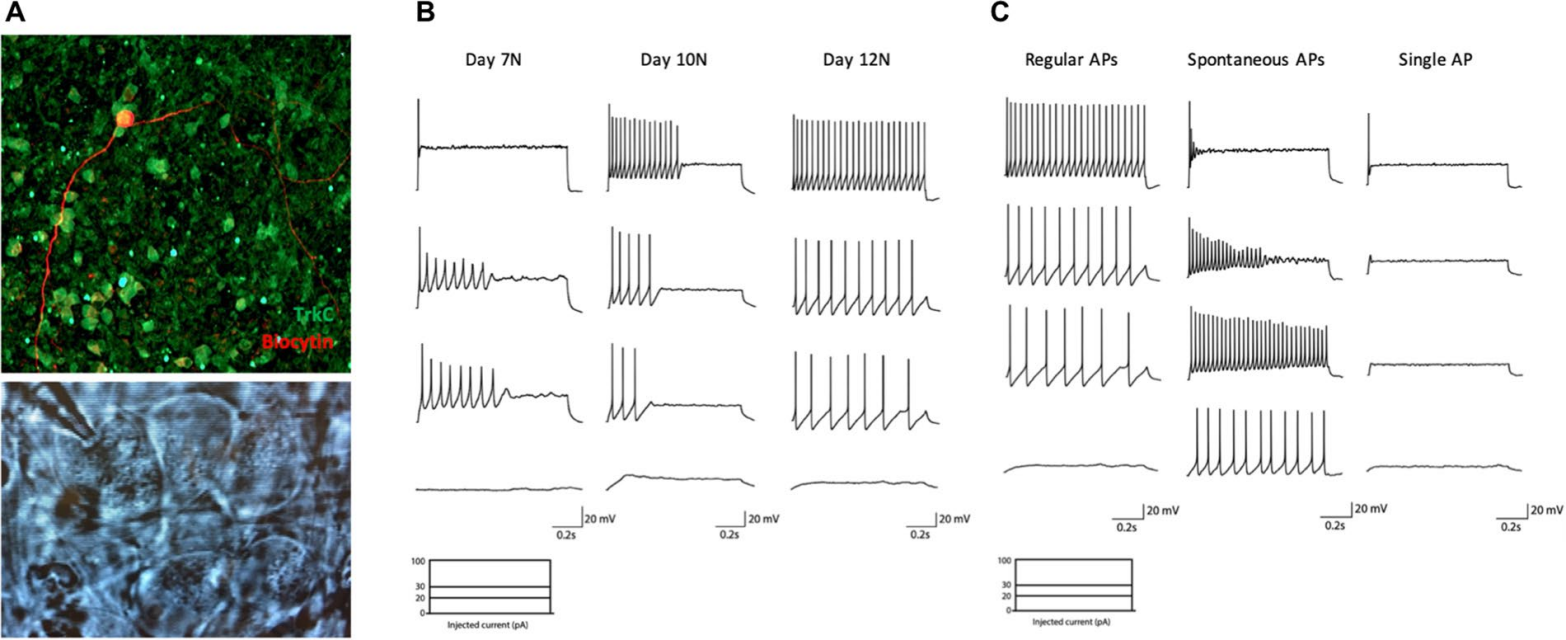
(**A**) (Up) Representative image of biocytin-filled TrkC^+^ neuron. (Down) Representative image of neurons identified with a 63x water immersion objective and an infrared CCD camera during electrophysiological recording. They showed the typical morphology of large sensory pseudo-unipolar neurons. Time course analysis of electrophysiological excitability of differentiated neurons. **(B)** Representative traces of current-clamp recordings in cells at day 7 (left) 10 (middle) or 12 (right) of treatment with neurotrophic factors (days 15, 18, 20 of differentiation), in response to depolarizing current injection steps at 0 pA (lower trace) 20, 30 and 100 pA (higher trace). **(C)** Representative traces of current-clamp recordings in cells at day 12 of treatment with neurotrophic factors, in response to 0 (lower traces) 20, 30 and 100 pA (higher traces) of depolarizing current injection steps. Three different responses were observed: regular APs firing with firing frequency increasing for higher current injections (left); spontaneous activity with rapid accommodation following increasing step current injections (middle); single AP in response to higher injecting currents (right).

Whole-cell patch-clamp recordings were performed to characterize the functional maturity of iPSC-derived neurons. A time course analysis of the electrophysiological excitability of these neurons in current clamp configuration (depolarizing current injection steps from 0 pA to 100 pA) was first performed in order to assess the timing of functional maturation. This analysis started on the 7th day of treatment with neurotrophic factors (day 15), when cells are already organized in clusters and show a mature morphological phenotype (Fig. 8A), and was pursued at day 10 and 12 of treatment with neurotrophic factors (day 18 and 20). The majority of neurons patched at the first stage showed only one AP at the beginning of the depolarizing current step, with just a few of them being able to generate a short train of low amplitude APs with rapid accommodation for increasing currents. Three days after, neurons exhibited an improved spiking profile, but were still characterized by a rapid accommodation in the majority of cases (Fig. 8B).

Finally, at day 20 (N = 15), it was possible to observe three different firing behaviors in the cultures (Fig. 8C): some neurons were capable of firing and sustaining regular APs in response to depolarizing current injections, with firing frequency increasing in response to higher currents; some neurons exhibited spontaneous AP firing but rapid accommodation following increasing step current injection; finally, some neurons were able to generate a single AP at the beginning of the depolarizing step in response to higher injecting currents. On average, recorded neurons exhibited a resting membrane potential of −62,75 ± 0,7493 mV.

Intrinsic passive properties of differentiated neurons at day 20 were obtained in voltage clamp configuration recording. Membrane resistance was 1963 ± 385.0 MΩ. The average capacitance was 29,65 ± 1,764 pF and the membrane time constant τ was 51.29 ± 6.35 ms.

### Differentiation of FRDA iPSCs

All three FRDA iPSC lines and differentiated sensory neurons maintained GAA repeat expansions in the *FXN* gene (not shown) and had repressed *FXN* expression (Fig. 4H). They could differentiate similarly to control cells and give rise to very similar mixed cultures of sensory neurons. FRDA neurons that survived beyond also had similar neurophysiological properties as control neurons. All three FRDA lines showed a lower expression of PV and RUNX3, both proprioceptor-specific markers, starting on day 13 (Figs. 2G and 4F), when the final segregation of proprioceptive neurons from TrkB^+^/TrkC^+^ lineage was expected to be completed. Differences for other markers were less consistent. In particular, Trk receptors were similarly expressed by control and FRDA lines and only two FRDA lines showed lower levels of the proprioceptor marker vGLUT1.

## Discussion

Numerous protocols have been developed for the generation of primary sensory neurons from pluripotent stem cells. However, they either do not enrich for a specific sensory neuron subtype or they focus on the generation of nociceptive neurons for the study of pain-related disorders. We developed a method to differentiate iPSCs into a primary sensory neuron population showing a substantial enrichment for proprioceptors, which can be FACS-purified for experiments as transcriptome analysis, including plate-based deep single-cell profiling^23^.

The cellular behavior and the pathways activated in the *in vitro* differentiation process resemble those observed during DRG development *in vivo*. We could robustly induce the transcription factors involved in sensory neurogenesis, driving the differentiation of iPSCs into cells expressing specific markers of DRG neurons. Furthermore, differentiated neurons could establish mature networks in just three weeks after plating, which is much faster than what is usually observed with iPSC-derived neurons. The specific timing of treatment of iPSCs with inhibitory factors was able to induce the rapid acquisition of a sensory precursor phenotype, while the combination of neurotrophic factors used in the stage of final differentiation and maturation allowed the generation of a high proportion of sensory neurons of the TrkB^+^/TrkC^+^ lineage. Nociceptive neurons depending on NGF for their survival were largely removed from the final culture, as this neurotrophin was only briefly added at low concentration. Minimal residual expression of TrkA in mature cultures was probably due to the activity of NT-3, which is able to bind, even if with low affinity, also to TrkA and TrkB. We used a high concentration of NT-3 and a low concentration of BDNF to favor final differentiation into TrkC^+^ proprioceptive rather than TrkB^+^ mechanoreceptive neurons. We obtained this way a substantial enrichment for TrkC^+^ cells, which represented 70% of the final cultures. However, we still obtained a substantial proportion (50%) of TrkB^+^ cells. These may in part be double positive neurons that co-express TrkB and TrkC. For this reason, we set up a FACS purification procedure to specifically collect cells with high TrkC expression, almost all of which are expected to be proprioceptors. Because this procedure damages neurons by severing their axons, it is appropriate for specific assays, like gene expression profiling, that do not require to put cells back in culture. For the same reason, FACS was performed before the final differentiation stage, when a very dense meshwork of neurites develops in the cultures, possibly explaining the still relatively high proportion of double TrkB^+^ and TrkC^+^ cells.

We demonstrated that this protocol can be used to differentiate iPSCs from patients carrying a genetic mutation leading to pathology affecting proprioceptive neurons, the GAA repeat expansion causing FRDA by repressing *FXN* gene expression. While we confirmed that *FXN* gene expression was decreased in FRDA cell lines, consistent with levels found in FRDA patients, we could not identify obvious maturation or survival defects in the differentiated cells. However, some suggestive differences emerged, in particular reduced induction of the proprioceptor-specific transcription factor RUNX3 and lower expression of PV in differentiated cultures at day 20. Furthermore, though FXN expression was slightly lower in the overall differentiated cultures than in iPSCs from both control and FRDA lines, it was higher in flow sorted TrkC^+^ cells, confirming in human cells the previous observation in the mouse that most FXN in DRGs comes from proprioceptors^15^, hinting to a possible cause of their specific vulnerability in FRDA. The availability of FRDA iPSC-derived primary proprioceptive neurons will eventually allow to fully characterize their cellular phenotype.

Finally, though we developed this model because of our interest in translational research in FRDA, it is of obvious interest to investigate many other conditions affecting proprioceptors, by using iPSC carrying relevant genetic mutations or by exposing cells to toxins and autoantibodies.

## Supplementary information

Supplementary Tabe 1.

## References

[1] Soldner F, Jaenisch R (2018). Stem Cells, Genome Editing, and the Path to Translational Medicine. Cell.

[2] Pandolfo M, Manto M (2013). Cerebellar and Afferent Ataxias. Continuum Lifelong Learn Neurology.

[3] Marmigère F, Ernfors P (2007). Specification and connectivity of neuronal subtypes in the sensory lineage. Nat Rev Neurosci.

[4] Hippenmeyer S (2005). A Developmental Switch in the Response of DRG Neurons to ETS Transcription Factor Signaling. Plos Biol.

[5] Poliak S, Norovich AL, Yamagata M, Sanes JR, Jessell TM (2016). Muscle-type Identity of Proprioceptors Specified by Spatially Restricted Signals from Limb Mesenchyme. Cell.

[6] Raible, D. W. & Ungos, J. M. Neural Crest Induction and Differentiation. 170–180, 10.1007/978-0-387-46954-6_10 (2006).

[7] Raible DW, Ungos JM (2006). Specification of sensory neuron cell fate from the neural crest. Adv Exp Med Biol.

[8] Ma Q, Fode C, Guillemot F, Anderson DJ (1999). NEUROGENIN1 and NEUROGENIN2 control two distinct waves of neurogenesis in developing dorsal root ganglia. Gene Dev.

[9] Hempstead BL (2014). Neurotrophic Factors. Handb Exp Pharmacol.

[10] Chambers SM, Mica Y, Lee G, Studer L, Tomishima MJ (2016). Dual-SMAD Inhibition/WNT Activation-Based Methods to Induce Neural Crest and Derivatives from Human Pluripotent Stem Cells. Methods Mol Biology Clifton N J.

[11] Koeppen AH, Ramirez RL, Becker AB, Mazurkiewicz JE (2016). Dorsal root ganglia in Friedreich ataxia: satellite cell proliferation and inflammation. Acta Neuropathologica Commun.

[12] Koeppen AH, Mazurkiewicz JE (2013). Friedreich ataxia: neuropathology revised. J Neuropathology Exp Neurology.

[13] Yandim C, Natisvili T, Festenstein R (2013). Gene regulation and epigenetics in Friedreich’s ataxia. J Neurochem.

[14] Schmucker S (2011). Mammalian frataxin: an essential function for cellular viability through an interaction with a preformed ISCU/NFS1/ISD11 iron-sulfur assembly complex. Plos One.

[15] Piguet F (2018). Rapid and Complete Reversal of Sensory Ataxia by Gene Therapy in a Novel Model of Friedreich Ataxia. Mol Ther.

[16] Hick A (2013). Neurons and cardiomyocytes derived from induced pluripotent stem cells as a model for mitochondrial defects in Friedreich’s ataxia. Dis Model Mech.

[17] Igoillo-Esteve M (2015). Unveiling a common mechanism of apoptosis in β-cells and neurons in Friedreich’s ataxia. Hum Mol Genet.

[18] Codazzi F (2016). Friedreich ataxia-induced pluripotent stem cell-derived neurons show a cellular phenotype that is corrected by a benzamide HDAC inhibitor. Hum Mol Genet.

[19] Lai J-I (2019). Transcriptional profiling of isogenic Friedreich ataxia neurons and effect of an HDAC inhibitor on disease signatures. J Biol Chem.

[20] Soragni E (2014). Epigenetic therapy for Friedreich ataxia. Ann Neurol.

[21] Ku S (2010). Friedreich’s ataxia induced pluripotent stem cells model intergenerational GAA⋅TTC triplet repeat instability. Cell Stem Cell.

[22] Eigentler, A., Boesch, S., Schneider, R., Dechant, G. & Nat, R. Induced Pluripotent Stem Cells from Friedreich Ataxia Patients Fail to Upregulate Frataxin During *In Vitro* Differentiation to Peripheral Sensory Neurons. *Stem Cells Dev* **22**, (2013). 10.1089/scd.2013.0126 23879205

[23] Picelli S (2013). Smart-seq. 2 for sensitive full-length transcriptome profiling in single cells. Nat Methods.

